# Investigation of the Preparation, Corrosion Inhibition, and Wear Resistance of the Chromized Layer on the Surfaces of T9 and SPCC Steels

**DOI:** 10.3390/ma15227902

**Published:** 2022-11-09

**Authors:** Sainan Liu, Jing Yang, Xiao Liang, Yangyang Sun, Xiaojun Zhao, Zhenyang Cai

**Affiliations:** 1 School of Minerals Processing and Bioengineering, Central South University, Changsha 410083, China; 2 School of Materials Science and Engineering, Central South University, Changsha 410083, China

**Keywords:** carbon steel, chromizing, coating, corrosion inhibition, wear resistance

## Abstract

To improve the corrosion inhibition and wear resistance of materials, the pack cementation method was used to prepare chromized coatings on the surfaces of high-carbon T9 steel and low-carbon SPCC steel. The results showed the formation of a uniform and dense double-layer structure with a thickness of ~10 μm on the surfaces of two different types of steel. The coating layer for T9 steel was mainly composed of Cr_23_C_6_ and Cr_7_C_3_, while that for SPCC steel was mainly composed of Cr_23_C_6_ and Fe–Cr solid solution. Additionally, both of the steels showed different hardness distributions. The hardness measurements of the outer layers of the T9 steel and SPCC steel were ~1737.72 HV and 1771.91 HV, while the hardness values of the secondary layers were 1378.31 HV and 448.52 HV, respectively. The polarization curves in 3.5 wt.% NaCl solution demonstrated the better corrosion resistance of the chromized coating. Chromizing increased the corrosion potential by ~0.2 V and reduced the corrosion current density by one order of magnitude. Under the presence of an 8 N load, the friction factor before and after the chromizing of T9 steel was about 0.69, and the mass wears were 2 mg and 0.6 mg, respectively. Meanwhile, the friction factor of the SPCC steel before and after chromizing was about 0.73, with respective mass wears of 2 mg and 2.9 mg. The wear resistance of T9 steel after chromizing was superior, but it became worse after chromizing for the SPCC steel.

## 1. Introduction

T9 carbon tool steel and SPCC low-carbon cold-rolled steel have wide applications in manufacturing industries [1,2]. Long-term durability and stable performance of steel are essential for industrial applications. However, T9 and SPCC steels both undergo corrosion and suffer from wear failures when exposed to corrosive environments and strong wear conditions in the long term [3,4], thus leading to safety accidents and even economic losses. Therefore, with respect to the broadening of application territories, improving the corrosion and wear resistance of T9 steel and SPCC steel is of great significance. Chromium stainless steel, which can be formed by the addition of chromium to steel matrices, has good corrosion and wear resistance due to the presence of hard chromium [5,6,7], but it is expensive and difficult to process. In such scenarios, surface modification [8,9] is an effective method for improving the corrosion and wear resistance of steel, and transition metal carbides or nitride layers can be produced through surface treatments [10,11].

Over the past decade, a variety of surface modification methods, such as electroplating [12,13], plasma spraying [14,15], salt baths [16,17], magnetron sputtering [18,19], laser cladding [20,21,22], the sol–gel method [23,24], fluidized beds [25,26], and pack cementation [27,28,29], have been developed. In spite of specific advantages, these technologies also have limitations, such as the requirement for expensive and complex equipment and high-vacuum conditions, weak adhesion of the formed coatings, serious environmental pollution, etc. As the pack cementation method involves powder landfill, it can deal with samples with complex shapes and inner holes. Moreover, due to the in situ formation of wear-resistant layers, such as chromium carbide through high-temperature chemical reactions, the coating and the steel substrate are closely metallurgically bonded due to the high bonding strength. Thus, a coating with excellent wear resistance and corrosion resistance can be prepared by pack cementation. If it is only used to improve the corrosion resistance of steel, its cost performance is not as good as that of electrochemical chromium plating and other methods. However, if corrosion resistance, wear resistance, and excellent bonding performance must be taken into account, the pack cementation method is one of the methods with the highest cost performance because of its low equipment cost, recyclable embedded materials, simple process system, and low cost [30,31]. Additionally, reports on the effect of chromizing on the surface of carbon steel and its related properties are already available [32,33]. However, the differences in corrosion and wear resistance between high-carbon steel and low-carbon steel after chromizing have not been studied. Therefore, a comprehensive study on the influence of surface chromizing on the corrosion and wear resistance of high-carbon steel and low-carbon steel is necessary.

In this work, the surface chromizing of high-carbon steel (T9) and low-carbon steel (SPCC) was carried out. During chromizing, the carbon in the steel diffuses to form a deposited layer of carbide-forming elements that then reacts with the carbide-forming elements in the layer. This results in the formation of a metallurgical bond between the carbide and the sample surface. Depending on the carbon content, different types of carbide coatings are formed on the surfaces of steel. The corrosion behavior was revealed in typical corrosive media, along with the friction and wear performance, under reciprocating friction conditions to elucidate the relationship between the carbon content and the corrosion and wear resistance of the materials.

## 2. Experimental Materials and Methods

### 2.1. Experimental Materials

For the preparation of the chromized layer, T9 steel (the international standard organization (ISO) standard steel grade is TC90) and SPCC steel (cold-rolled carbon steel, the chemical composition and mechanical properties of which are similar to those of Q195 and Q215A) were selected as base materials, and the chemical compositions (mass fraction) are shown in Table 1.

### 2.2. Experimental Method

**Figure 1:**
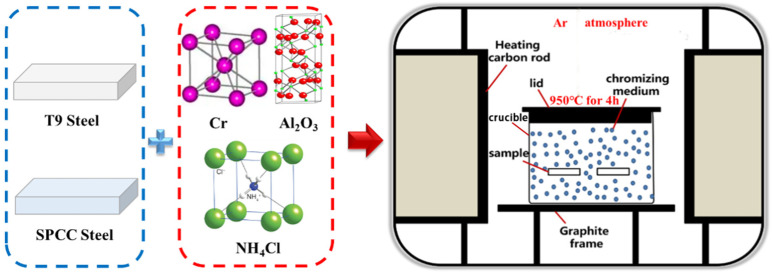
Schematic diagram of the chromizing device.

The solid powder pack cementation method was adopted for the chromizing process, and the device is shown in Figure 1. The dimensions of the machined sample were 25 mm × 10 mm × 3 mm. In order to remove the surface oxide layers and machining traces, water-abrasive paper was used to polish the sample, and the sample was then ultrasonicated with anhydrous ethanol, followed by drying. The chromizing powder was composed of a chromium donor, filler, and activator. Pure chromium powder was used as the chromium donor. Al_2_O_3_ powder was used as the filler, while NH_4_Cl was the activator. The chromizing powder was prepared with a certain ratio and placed in a mixer. The well-mixed chromated powder was added to the crucible. The steel sample was evenly buried in the chromated powder dispersed in the crucible. Then, the crucible was closed and placed into a high-temperature furnace, where it was heated at 950 ℃ for 4 h under an argon atmosphere. During this process, Cr reacted with NH_4_Cl to produce reactive chromium atoms and counteracted the carbon atoms in the substrate to form Cr–C compounds. Afterward, the carbon in the substrate diffused outward to form a new phase, and the chromium atoms further replaced the substrate crystal space to form an Fe–Cr solid solution. The Cr–C compound and Fe–Cr solid solution layer that formed during this process due to diffusion was called the “infiltrated layer”. After cooling to room temperature, the sample was removed and underwent ultrasonic treatment for the removal of the adhesive. The sample was placed in an alcohol solution containing 4% (volume fraction) nitric acid for a certain amount of time, and then alcohol was used for cleaning with ultrasonic assistance.

### 2.3. Material Characterization

The phase composition of the infiltrated layer was analyzed using a D/max 2550 automatic X-ray diffractometer (XRD, Rigaku DX-2500, Rigaku, Tokyo, Japan) using a Cu target at 40 kV, and the scanning angle (2θ) was 20–90°. Three-dimensional imaging of the surface-infiltrated layer of the sample was conducted using an LSM700 laser confocal microscope (LSCM, LSM700, Zeiss, Jena, Germany) with an imaging range of 600 μm × 600 μm. The microstructural morphology of the surface was observed by employing a MIRA3 TESCAN-type field emission scanning electron microscope (SEM). A JXA-8230R electron probe microanalyzer (EPMA, JEOL JXA-8230, JEOL Ltd., Tokyo, Japan) equipped with WDS was used to scan the micro area of the coating cross-section and to determine the elemental distribution of the composite coating. To obtain cross-sectional samples for testing, the T9 steel and SPCC steel samples with coatings were wire-cut into small pieces of 5 mm × 10 mm × 3 mm and were then ultrasonically cleaned and embedded in resin. After sanding with 200, 400, 600, 800, 1000, 1500, and 2000 mesh sandpaper, the samples were polished with W2.5 and W1.5 polishing paste.

To refrain from artificial reading errors in the test process affecting the accuracy of the hardness value, a computer-connected nanoindenter was selected for hardness testing, and a UNHT nanoindenter was used to test the hardness distribution of the sample from the infiltration layer to the inside of the substrate. The loading speed and loading time were 40 mN/min and 10 s, respectively. A multi-autolab M204 electrochemical workstation was used to test the polarization curves of the samples. A three-electrode system employing 3.5 wt.% NaCl solution as the corrosive medium was used. For the polarization curve measurements, the matrix was cut into 10 mm × 10 mm for chromizing; then, the resin and wire were used to form the chromized sample into a standard sample for the polarization test. Additionally, the balance time was 1 h. A UMT-3-type reciprocating friction tester was used to test the friction and wear performance of the samples. The friction pair was composed of Si_3_N_4_ ceramic balls with a diameter of 9.5 mm, the load was 8 N, the test time was 30 min, and the speed was 240 rpm.

## 3. Results and Analysis

### 3.1. XRD Analysis

**Figure 2:**
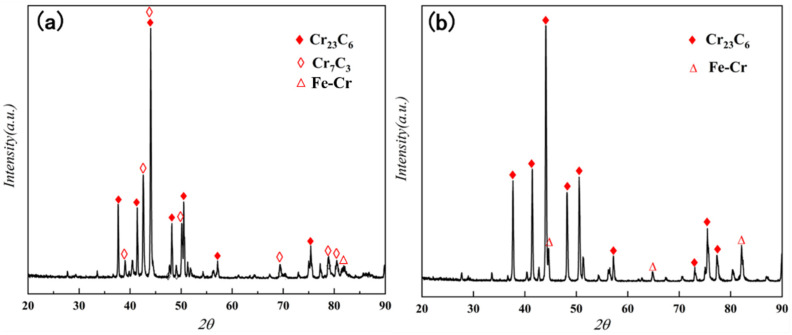
Surface XRD patterns of (**a**) chromized T9 steel and (**b**) chromized SPCC steel.

The XRD patterns of both of the chromized steels are shown in Figure 2. The surface layer of the chromized T9 steel was mainly composed of Cr_23_C_6_ and Cr_7_C_3_ phases. T9 steel showed a higher carbon content, and Cr appeared to be a strong carbide-stabilizing element. At high temperatures, outward diffusion of C in the substrate occurred. This resulted in the formation of a carbon-rich zone on the surface, and the combination of some of the Cr with C resulted in the Cr_23_C_6_ phase. With the continuous enrichment of the C elements on the surface, part of the Cr_23_C_6_ phase was transformed into the Cr_7_C_3_ phase [34]. However, the surface layer of the chromized SPCC steel was mainly composed of the Cr_23_C_6_ phase and Fe–Cr solid solution. Due to the relatively low carbon content of SPCC steel, only the Cr_23_C_6_ phase formed on the surface. The lack of sufficient carbon potential prevented the transformation of the Cr_23_C_6_ phase into the Cr_7_C_3_ phase. At elevated temperatures, the Cr and Fe atoms inter-diffused to form an Fe–Cr solid solution.

### 3.2. Confocal 3D Imaging Analysis

**Figure 3:**
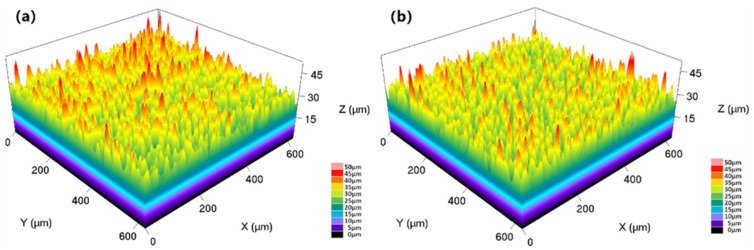
Three-dimensional images of the (**a**) chromized T9 steel surface and (**b**) chromized SPCC steel surface.

The three-dimensional images of the surface acquired by the laser confocal microscope are shown in Figure 3. The surface roughness (Ra) values of T9 and SPCC steel after chromizing were 4.02 μm and 3.52 μm, respectively. The surface roughness of chromized SPCC steel was slightly lower than that of the chromized T9 steel.

Moreover, within a microscopic regime, the surface of the samples after chromizing showed a uniform, but locally clustered, morphology, which was determined by the characteristics of the solid powder employed in the chromizing process in relation to the pretreatment process of the sample. When the penetrating agent and the sample came into contact, the diffusion and enrichment of the reactive gas in the gap between the sample and powder led to the preferential growth of the surface-infiltrating layer.

### 3.3. Elemental Analysis of the Infiltrating Layer

**Figure 4:**
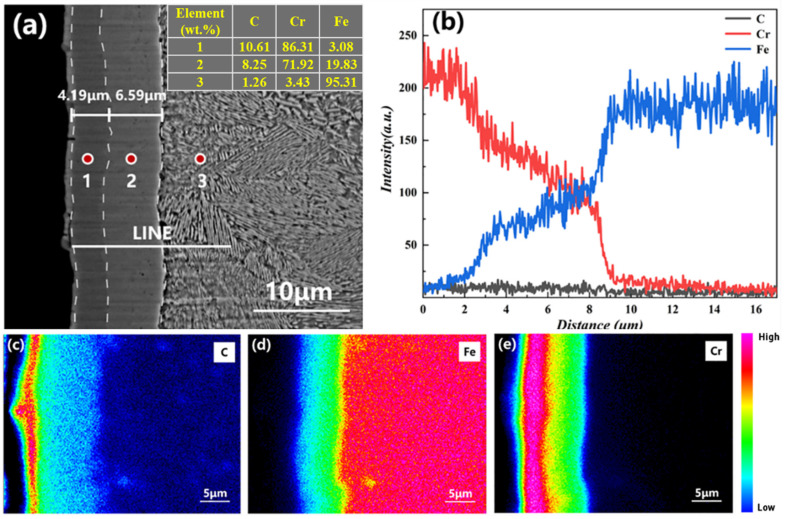
(**a**) Cross-section morphology of the chromized T9 steel. (**b**) Elemental distribution trend along the straight line shown in (**a**): surface scanning of the (**c**) C, (**d**) Fe, and (**e**) Cr distributions.

The cross-sectional morphology of the coating is shown in Figure 4a. The chromized layer of T9 steel was composed of inner and outer layers, which was obvious from the contrast. The thickness of the outer layer was ~4.19 μm, while the thickness of the secondary layer was ~6.59 μm. Overall, there were no obvious defects. As obtained from the elemental distribution shown in Figure 4b, with increasing distance, there was a stepwise alteration in the concentrations of Cr and Fe. The outer layer had the highest Cr content, which showed a decreasing trend in the secondary layer along with an increase in the Fe content. Equilibrium was reached at the junction between the coating and the substrate. With deeper penetration into the substrate, there was a sharp decrease in the elemental content of Cr, while the concentration of Fe reached its maximum value. The C content was slightly higher in the infiltrating layer compared with that of the substrate. These results are consistent with the inset table in Figure 4a, which provides the quantitative analysis of the chemical composition distributed on the surface by WDS.

The distributional characteristics of different coating elements can be obtained from the surface mapping of the cross-sectional area of the coating (Figure 4c–e). From the outside to the inside, the contents of the C and Cr elements gradually decreased, while the opposite trend was observed for Fe. This may be attributed to the deposition of the infiltration agent on the surface of the infiltrating layer during inclusive infiltration. The highest contents of C and Cr were found in the outer layer. In the combined XRD and elemental distribution analysis, the outer layer of the infiltrating layer was found to be composed of Cr_7_C_3_ and Cr_23_C_6_ phases. The lower contents of the elements C and Cr and a higher Fe content in the secondary layer indicated that ferrochrome carbide was the main component of the outer layer in the infiltrating layer. The lower C content in the substrate compared with that in the infiltrating layer suggested the diffusion of C to the surface layer at high temperatures.

**Figure 5:**
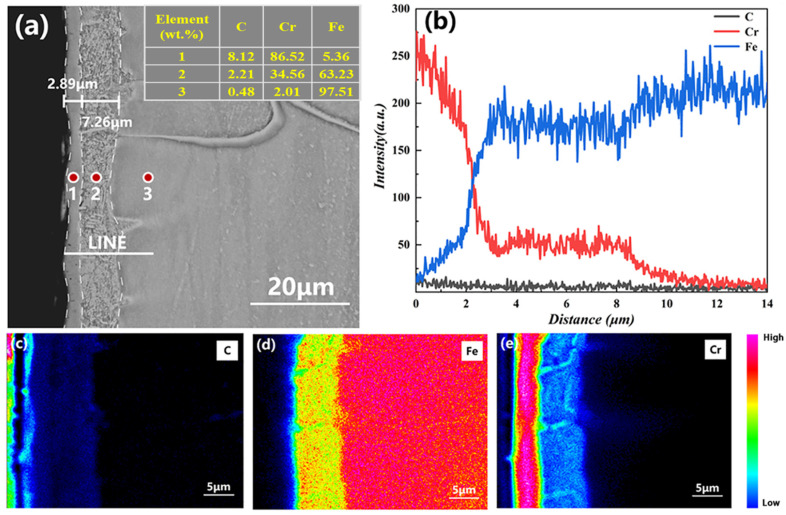
(**a**) Cross-sectional morphology of the chromized SPCC steel. (**b**) Elemental distribution trend along the straight line shown in (**a**); surface scanning of (**c**) C, (**d**) Fe, and (**e**) Cr distributions.

**Figure 6:**
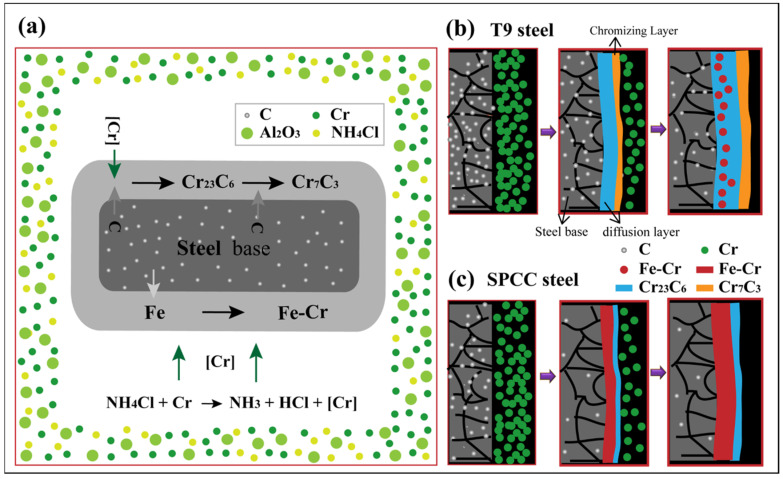
(**a**) Schematic diagram of chromizing reaction of steel matrix; chromizing process diagram of (**b**) T9 and (**c**) SPCC steels.

Figure 5a shows the cross-sectional morphology of the chromized SPCC steel coating, as well as two chromized layers. Here, the sample was also stratified by the different contrast. The thickness values of the outer layer and secondary layer were ~2.89 μm and ~7.26 μm, respectively. No apparent defects were found. A stepwise alteration trend with increasing distance for the concentrations of Cr and Fe can be seen in Figure 5b, similar to that in T9. The highest Cr content was observed in the outer layer, which then dropped sharply in the second layer. At the same time, the Fe content started to increase, and equilibrium was reached at the junction of the outer layer and the secondary layer. The secondary layer showed a significantly higher Fe content compared with that of Cr, and the infiltrating layer had a slightly higher C content than that of the substrate. These results are consistent with the inset table in Figure 5a, which depicts the quantitative analysis of the chemical composition distributed on the surface by WDS. The results of mapping analyses for different elements on the cross-sectional area of the coating section are shown in Figure 5c–e. There was a gradual decrease in the C and Cr contents from the outside to the inside, while the Fe content gradually increased from the outside to the inside. The highest C and Cr contents were observed in the outer layer. XRD analysis showed that the outer layer of the infiltrating layer consisted of the Cr_23_C_6_ phase. A significantly higher Fe content was found in the secondary layer compared with C and Cr, indicating that the outer layer of the infiltrating layer was composed of Fe–Cr solid solution. The difference between Figure 4 and Figure 5 is that the hardness of the matrix is different, resulting in different polishing and grinding values. However, what is more important is that different substrates had different sensitivity levels to the corrosion solution, which led to different corrosion depths and corrosion morphologies. Additionally, according to the depth distribution profiles of chromium in Figure 4 and Figure 5, the thickness of the near-surface layer forming the diffraction pattern in Figure 2 may have been 5–10 μm. Combined with the XRD and EDS results of T9 and SPCC steels, the chromizing process is shown in Figure 6. The pure chromium powder reacted with the activator NH_4_Cl to obtain activated chromium atoms. The activated chromium atoms first reacted with the carbon atoms on the surface of the steel substrate to form Cr_23_C_6_. The carbon in the substrate continued to diffuse to the surface, which promoted the transformation of Cr_23_C_6_ into Cr_7_C_3_. Compared with T9 steel, SPCC steel had a lower carbon content and only formed a thinner chromium carbide layer. Driven by the low carbon content and high temperatures, chromium atoms replaced crystal spaces in the austenite phase in the SPCC steel matrix to form the Fe–Cr solid solution.

### 3.4. Nanoindentation Microhardness Analysis

**Figure 7:**
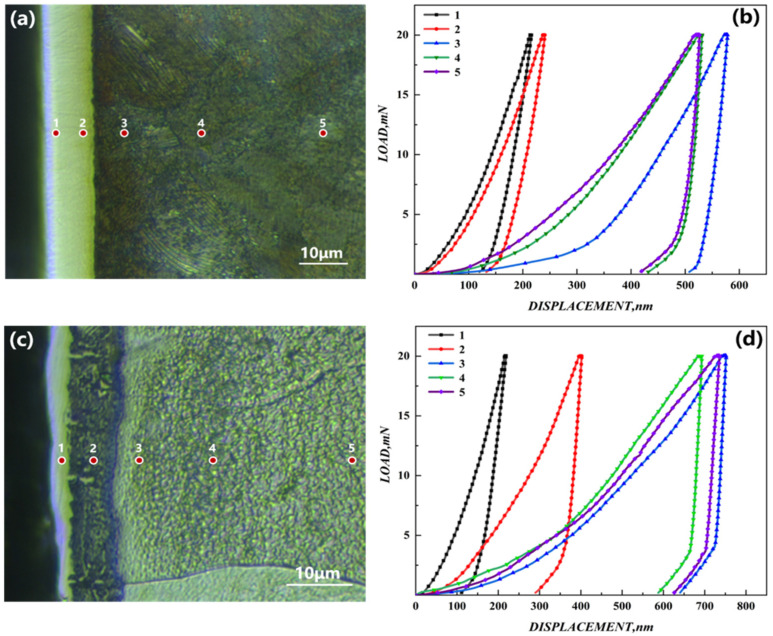
(**a**) Schematic diagram of the indentation of chromized T9 steel; (**b**) load–press-depth curves of chromized T9 steel; (**c**) schematic diagram of indentation of chromized SPCC steel; (**d**) load–press-depth curves of chromized SPCC steel.

The indentation images and load–press-depth curves of chromized T9 and SPCC steel are shown in Figure 7, with the micro-Vickers hardness (HV) of the chromized T9 and SPCC steels at different depths from the surface being presented in Table 2.

The hardness of the outer infiltrating layer of chromized T9 steel was 1737.72 HV. According to XRD analysis and the energy spectrum, the phase consisted of Cr_7_C_3_ and Cr_23_C_6_, which resulted in maximum hardness. At a depth of 6 μm from the surface, the hardness of the secondary infiltrating layer was reduced to 1378.31 HV. The phase consisted of ferrochrome carbide. With increasing depth, the hardness decreased sharply compared with the outer layer, with the substrate having a hardness of 219.29. For chromized SPCC steel, the hardness of the outer layer was 1771.91 HV. At the depth of 6 μm, Fe–Cr solid solution was the main phase, which resulted in a rapid decrease in the hardness to 448.52 HV that decreased continuously to a value of 131.09 HV. The decreasing trend of the hardness from the surface-infiltrating layer to the internal substrate for both the chromized T9 and SPCC steels showed gradient behavior, which was closely related to the gradient distribution of the elements in the chromized layer. When the infiltrating layer formed, there was outward diffusion of carbon elements from the surface of the substrate, resulting in the formation of a decarburized zone and the slightly lower hardness of the substrate near the infiltrating layer compared with that of the core of the substrate.

### 3.5. Electrochemical Corrosion Test

**Figure 8:**
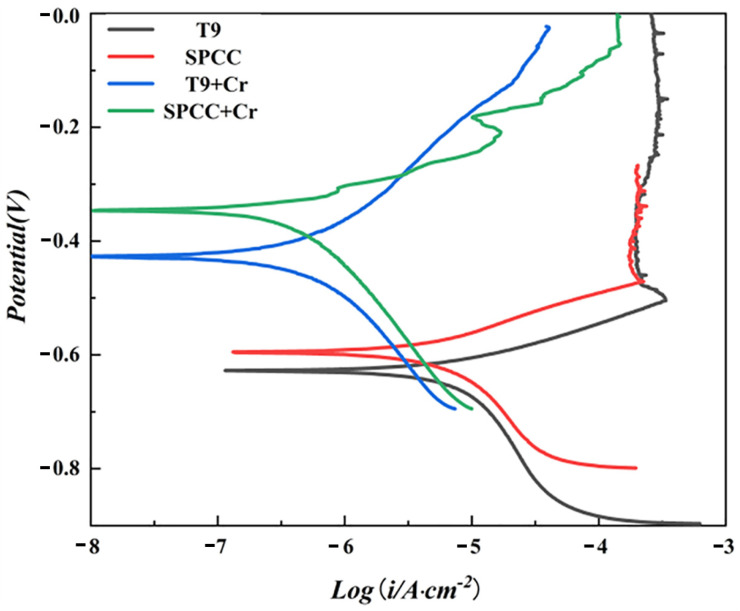
Polarization curves of the T9 steel, SPCC steel substrate, and the samples after chromizing in a 3.5 wt.% NaCl solution.

The self-corrosion potential (E_corr_) and corrosion current density (i_corr_) are often used to evaluate the corrosion resistance of coatings [35,36]. The improved corrosion resistance of various materials was characterized by increased self-corrosion potential and decreased corrosion current density. The polarization curves of the T9 and SPCC steel substrates in 3.5 wt.% NaCl solution before and after chromizing are shown in Figure 8.

**Figure 9:**
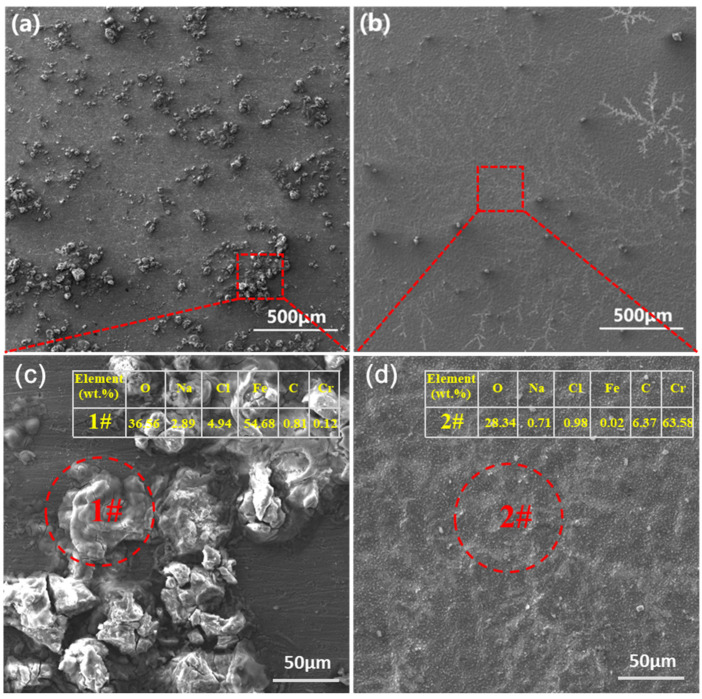
Surface morphologies of the (**a**) T9 steel substrate, (**b**) chromized T9 steel after immersion in a 3.5 wt.% NaCl solution for 120 h, (**c**) partial enlargedb image of (**a**) and element analysis of 1#, (**d**) partial enlargedb image of (**b**) and element analysis of 2#.

As shown in Figure 8, the self-corrosion potentials of the T9 and SPCC steel substrates were −0.63 V and −0.60 V, respectively, which, after chromizing, increased to −0.42 V and −0.35 V, respectively, leading to respective increases of 0.21 V and 0.25 V. Initially, the calculated i_corr_ values for the T9 steel and SPCC steel substrates were 4.04 × 10^−6^ and 3.03 × 10^−6^, respectively, as obtained from the linear fitting on the Tafel zone of the polarization curve. After chromizing, the i_corr_ values of T9 steel and SPCC steel decreased by one order of magnitude to reach the respective values of 3.05 × 10^−7^ and 2.26 × 10^−7^. The chromized layer was able to play a protective role against corrosion, significantly improving the corrosion resistance of the T9 steel and SPCC steel. The surface morphologies of T9 steel after immersion in 3.5 wt.% NaCl solution for 120 h with or without chromizing are shown in Figure 9.

After 120 h of immersion in a 3.5 wt.% NaCl solution, the surface of the T9 steel substrate showed the presence of a large number of nodular corrosion products (Figure 9a). However, under identical experimental conditions, the chromized T9 steel surface showed fewer surface corrosion products (Figure 9b). Composition analysis by WDS showed that a large number of iron oxides and more Cl ions formed on the surface of T9 steel (Figure 9c), while uniform corrosion-resistant chromium oxides formed on the surface of the chromized T9 steel, and the retention of Cl ions decreased significantly (Figure 9d). This proves that the chromized layer could effectively protect the substrate from being corroded in a saltwater environment.

### 3.6. Friction and Wear Performance Tests

**Figure 10:**
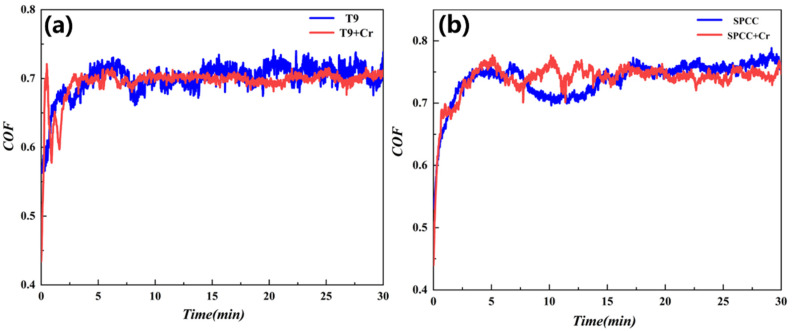
Variation curves of the friction coefficient: (**a**) T9 steel substrate and chromized T9 steel and (**b**) SPCC steel substrate and chromized SPCC steel.

Figure 10 shows the time-dependent relationship curves of the friction coefficients for T9 steel, the SPCC steel substrate, and the chromized samples. It can be seen that, in the initial running-in stage, for both steel substrates and the chromized samples, a sharp increase in the friction coefficients was observed with increasing sliding stroke. The friction coefficients of the chromized samples were not significantly different from those of the base materials, indicating no anti-friction effects for the chromized layer. Combined with the three-dimensional imaging of the surface, this may be due to the ubiquitous dispersion of many hard particles on the surface after the chromizing treatment, leading to an increase in the surface roughness.

**Figure 11:**
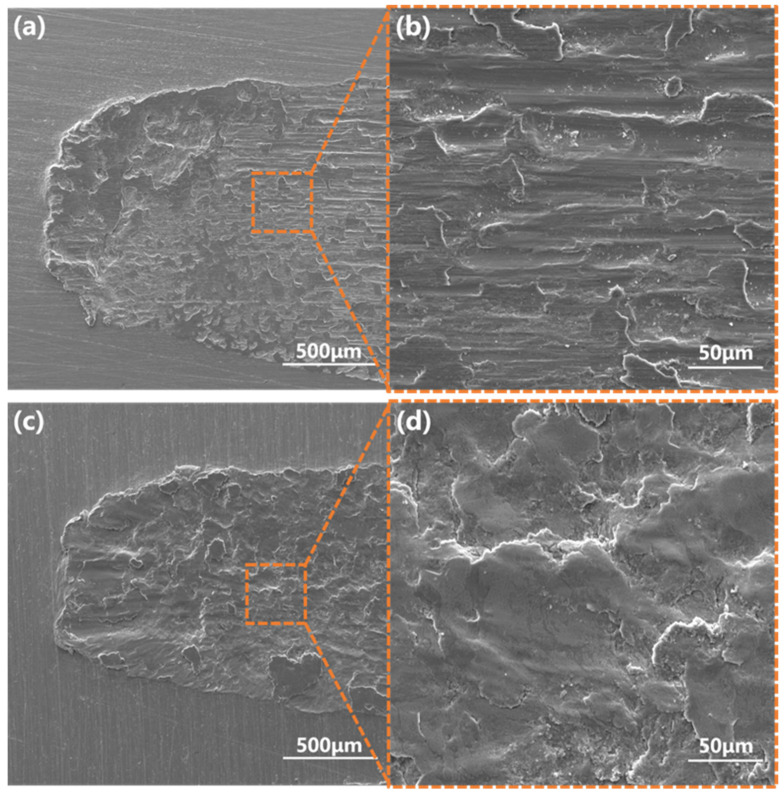
Morphologies of (**a**,**b**) T9 steel substrate and (**c**,**d**) SPCC steel after wearing.

For the T9 steel substrate, the friction coefficient in the running-in stage linearly increased to 0.68, which then subsequently jittered up and down from 0.65 to 0.75, as can be seen in Figure 10a, and the average friction coefficient was 0.6997. For the chromized sample, in the first three minutes, there were drastic fluctuations in the friction coefficient. Afterward, it became stable within the range of 0.68 and 0.73, with the average friction factor being 0.6937. The mass wear values of the base material and chromized samples were 2 mg and 0.6 mg, respectively, indicating that the wear resistance of T9 steel was significantly enhanced after the chromizing treatment. From Figure 10b, the friction coefficient of the SPCC substrate linearly increased to 0.65 in the running-in stage. Then, it jittered up and down from 0.69 to 0.79, with 0.737 being the average friction coefficient. As for the chromized sample, the friction coefficient linearly increased to 0.68 and then jittered up and down from 0.70 to 0.78, leading to an average friction factor of 0.7391. The mass wear values of the base material and chromized sample were 2 mg and 2.9 mg, respectively, indicating that the chromizing treatment had a poor effect on the wear resistance of SPCC steel. The wear scar morphology and electron microscope analysis indicated that a carbide layer formed after chromizing. The SPCC low-carbon steel was thin and quickly wore down during the abrasion process. Additionally, the ground hard carbide particles facilitated the wear of the substrate under the infiltrating layer. Figure 11 indicates the morphologies of the T9 and SPCC steel surfaces after wearing under different magnifications.

**Figure 12:**
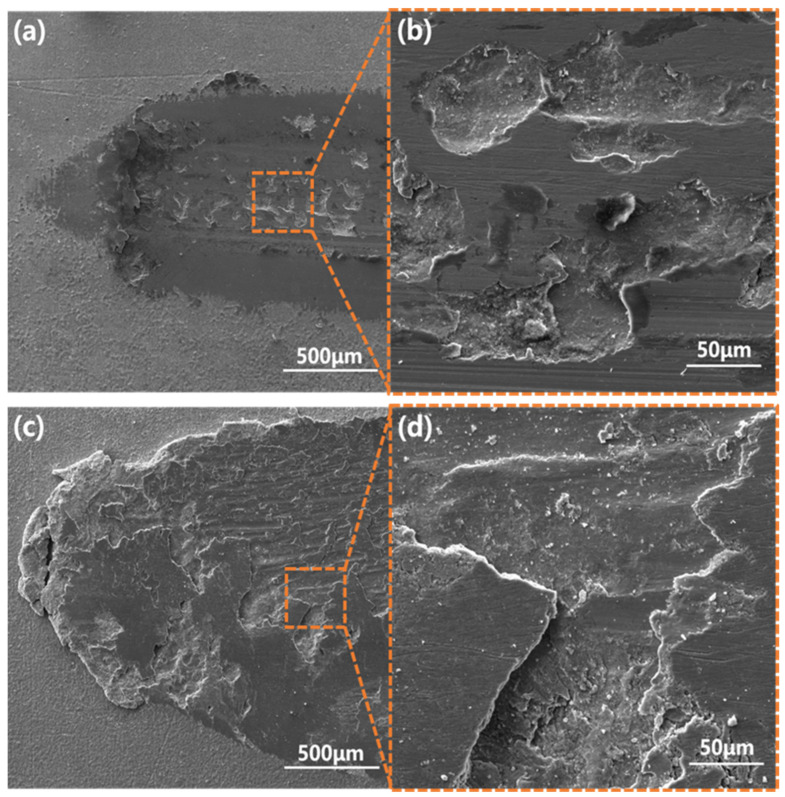
Wear scar morphologies of (**a**,**b**) chromized T9 steel and (**c**,**d**) chromized SPCC steel.

As shown in Figure 11a,b, after abrasion, the surface roughness of the T9 steel substrate increased, and the surface exhibited flaky adhesion pits and a large number of furrows. This was due to the occurrence of metal adhesion under friction with a grinding ball resulting in the appearance of adhesion points. In the presence of reciprocating friction, the metal on the surface of the material was torn to form abrasive particles, and under the action of plowing, a large number of furrows were formed. The wear mechanism consisted of adhesive wear and abrasive wear [37,38,39]. In Figure 11c,d, the SPCC steel substrate only showed adhesive wear, with no furrows being visible to the naked eye. This was due to the low hardness of SPCC steel; the wear debris that wore off did not form surface furrows, and the wear mechanism was adhesive wear. Figure 12 shows the wear scar morphologies of the chromized T9 and SPCC steels under different magnifications.

After chromizing, T9 only showed a small amount of adhesive wear, as can be seen in Figure 12a,b. The adhesive pits were small in size, and they were independent of each other. There was no wear on either side of the wear scar. Compared with the substrate, the wear resistance of the sample after chromizing was significantly improved, with adhesive wear being the wear mechanism. As shown in Figure 12c,d, the infiltrating layer in some areas of the SPCC steel remained intact after chromizing, while in other areas, a large number of furrows were formed. The abrasive wear phenomenon was more apparent. Combined with the scanning electron microscopic analysis, after chromizing the SPCC steel, a thin carbide layer was formed and the contact surface of the friction pair showed sliding friction. Due to periodic loading, the generation of large alternating stress in the contact area resulted in the occurrence of cracks, and fractures occurred in the weak parts of the surface, leading to the peeling off of the infiltrating layer and causing surface fatigue wear [40,41]. A large number of hard abrasive particles were formed after the peeling of the infiltrating layer, which accelerated the abrasive wear of the substrate under the infiltrating layer. Thus, the wear mechanism of SPCC steel after chromizing consisted of surface fatigue wear and abrasive wear.

## 4. Conclusions

The pack cementation method was used to prepare chromized coatings on the surfaces of high-carbon T9 steel and low-carbon SPCC steel, which resulted in a double-layer structure with a thickness of ~10 μm. For T9 steel, the outer layer was composed of Cr_7_C_3_ and Cr_23_C_6_ phases, with a hardness of 1737.72 HV, while ferrochrome carbide was the component of the secondary layer, with a hardness of 1378.31 HV. As for SPSS steel, the outer layer was composed of a Cr_23_C_6_ phase with a hardness of 1771.91 HV, and the secondary layer was composed of an Fe–Cr phase with a hardness of 448.52 HV. The self-corrosion potentials of the T9 steel and SPCC steel increased by 0.21 V and 0.25 V, respectively, due to chromizing, indicating that the chromized layer effectively improved the corrosion resistance of substrates. The friction factor of the T9 steel before and after chromizing was more or less similar, at about 0.69, while the mass wear values were 2 mg and 0.6 mg, respectively. The wear mechanisms changed from adhesive wear and abrasive wear prior to chromizing to only adhesive wear after chromizing. For SPCC, the friction factor before and after chromizing remained the same at ~0.73, and the mass wear values were 2 mg and 2.9 mg, respectively. The wear mechanism changed from adhesive wear prior to chromizing to surface fatigue wear and abrasive wear after chromizing.

Due to the relatively high carbon content of the T9 steel, a double-layer infiltrating layer composed of chromium carbide was formed. The uniform and dense hard coating effectively improved the corrosion resistance and wear resistance of the T9 steel. However, for SPCC steel, the carbon content decreased, which resulted in a thin outer chromium carbide layer, most of which consisted of the Fe–Cr solid solution of the secondary layer. The corrosion resistance of materials can be improved by a thinner chromium carbide layer, but it is prone to surface fatigue wear during friction processes, which can lead to material failure. Therefore, chromizing treatment cannot improve the wear resistance of low-carbon SPCC steel.

## Figures and Tables

**Table 1:** Chemical composition (mass fraction) of T9 steel and SPCC steel.

	C	Mn	P	S	Si	Cr	Ni	Cu	Fe
T9	0.8~0.9	≤0.50	≤0.03	≤0.03	≤0.35	≤0.20	≤0.25	≤0.30	Bal.
SPCC	≤0.15	≤0.60	≤0.100	≤0.02	-	-	-	-	Bal.

**Table 2:** Micro-Vickers hardness (HV) of the chromized T9 and SPCC steels at different depths from the surface.

Position		1	2	3	4	5
Depth (μm)		1.5	6	13	26	45
T9	1	1734.55	1374.92	221.43	252.35	256.89
	2	1739.26	1375.26	219.78	250.69	254.31
	3	1735.35	1382.07	215.48	246.48	257.67
	4	1740.84	1380.42	217.52	253.99	251.44
	5	1738.60	1378.88	222.24	255.84	255.84
	Average	1737.72	1378.31	219.29	251.87	255.23
SPCC	1	1773.65	445.42	134.83	154.24	147.73
	2	1768.58	447.28	131.06	152.58	149.64
	3	1769.82	449.60	127.94	147.33	144.92
	4	1774.64	451.56	130.69	149.86	148.67
	5	1772.86	448.74	130.93	152.74	145.84
	Average	1771.91	448.52	131.09	151.35	147.36

## Data Availability

The data presented in this study are available on reasonable request from the corresponding author.
